# Ecological and Geochemical Conditions for the Accumulation of Antioxidants in the Leaves of *Lathyrus maritimus* (L.) Bigel

**DOI:** 10.3390/plants9060746

**Published:** 2020-06-13

**Authors:** Pavel Maslennikov, Elena Golovina, Anastasia Artemenko

**Affiliations:** 1 Institute of Living Systems, Immanuel Kant Baltic Federal University, Universitetskaya Str., 2, 236040 Kaliningrad, Russia; art_anastasia@mail.ru; 2 Kaliningrad Institute of Economics, Karl Marx Str., 17, 236040 Kaliningrad, Russia; golowina@mail.ru

**Keywords:** Curonian Spit, *Lathyrus maritimus* Bigel, antioxidants, ascorbate, antioxidant activity, polyphenols, ascorbate peroxidase, catalase

## Abstract

The article explores how location affected the dynamics of accumulation of ascorbic acid (AC) and oxidized forms of AC—dehydroascorbic acid (DAA) and diketogulonic acid (DKGA) in beach pea during ontogenetic development. Our analysis focuses on research of the ecological and geochemical conditions growing of the plant on the Curonian Spit. The level of hydrogen peroxide and the activity of enzymes that break it down were analyzed. Antioxidant activity and the total concentration of phenolics were evaluated in the leaves of beach pea on the leeward and windward sides of the foredune. It was established that the level of AC, DAA, and DKGA was higher in the plants growing on the windward side of the foredune. A higher concentration of peroxy compounds, which stimulate the biosynthesis of antioxidant enzymes (catalase, ascorbate peroxidase), polyphenols, and other low molecular antioxidants (AOA) was observed in the leaves of these plants. The plants on the windward side enter phenological stages one or two weeks later than their counterparts on the leeward side of the foredune do. There was a generally negative correlation between the temperature of the soil and the accumulation of ascorbate system acids in the leaves of the studied plants (r = −0.46/(−0.68), *p* < 0.05). The accumulation of low molecular antioxidants and enzymes in beach pea suggests their adaptation to the adverse conditions of the windward side of the foredune.

## 1. Introduction

The way plants adapt to a changing environment depends on many physio-biochemical mechanisms, particularly, the efficiency of their antioxidant systems. The antioxidant system of a plant cell comprises various interconnected redox reactions that involve antioxidant enzymes and low-molecular metabolites. Under normal conditions and oxidative stress, antioxidant enzymes (superoxide dismutase, various peroxidases, catalase, and ascorbate–glutathione cycle enzymes) play an important role in maintaining safe levels of reactive oxygen species (ROS) in cells [1,2,3,4].

Today, studies into the mechanisms of plant adaptation to biotic stresses (salination, ultraviolet radiation, etc.) use either model plants that vary dramatically in resistance or transgenic plants with altered expression of the genes that encode antioxidant enzymes or the enzymes of biosynthesis and catabolism of low-molecular antioxidants [5]. However, the problem of biodiversity conservation concerns a broad range of wild plants. Having never experienced selection, wild plants may differ significantly from model plants as regards the constitutive or stress-induced levels of known adaptive reactions [5]. Studies of wild plants expand our knowledge of the general patterns of defense responses to stress and thus are key to developing biocoenosis conservation strategies. Survival limits for wild plants exposed to changing conditions have been studied insufficiently, particularly, as regards the defense response of plants. Another important aspect of research into the defense response of plants is the regulatory and compensatory mechanisms of the antioxidant system. Despite the above considerations and ample literature describing the functioning of antioxidant enzymes [3,6] and low-molecular metabolites [7,8,9], there is little information on the regulatory or compensatory mechanisms of the antioxidant system.

All the above lends a new urgency to studies into the plants that dominate the flora of the dunes on the Curonian Spit. Constantly exposed to adverse factors, these plants accumulate significant biomass. Thus, they can be considered ecological resilience champions. The mechanism of such resilience, however, has not been investigated.

One of these plants is beach pea. Beach pea (*Lathyrus maritimus* L.), a relatively unknown leguminous plant, grows along the sandy and gravel shorelines of Canada, Greenland, Siberia and Japan. The roots of the beach pea plant are nodulated by the nitrogen-fixing soil bacterium, *Rhizobium* under naturally growing conditions. Besides being a sand binder, due to the horizontally growing underground stems and roots, it is sometimes used as a fodder for cattle. Unlike other legumes, beach pea is not cultivated, in part, because its nutritional value and possible presence of toxicants have not been studied previously. In particular, the antioxidant properties of this plant remain poorly understood.

Ascorbic acid is a multifunctional compound. It regulates the activity of the enzymes that take part in cell division and expansion [10] during growth, vegetative and reproductive differentiation, water exchange, photosynthesis and respiration [11,12,13,14]. The consideration of ascorbic acid as an element of the AC $\begin{array}{c} ↔ \end{array}$ DAA → DKGA organic acid system makes it possible to establish the direction of the redox processes taking place in that system and to get a comprehensive picture of the role of ascorbic acid in plant metabolism under normal conditions and stress. Another insufficiently studied question is how antioxidant enzymes function during changes in the metabolism of low-molecular substances (for example, polyphenols) and how these changes affect the redox homeostasis of a cell. Analyzing the content of low-molecular antioxidants in wild plants in various nature reserves across the world is crucial for developing a biodiversity conservation program [2,4].

In this regard, the ecological and geochemical conditions of the formation of the beach pea antioxidant pool (TAC) were studied, the content of low-molecular antioxidants and antioxidant enzymes was analyzed depending on the growing conditions on the foredune. The dynamics of the accumulation of AC and oxidized forms of AC–DAA and DKGA during ontogenetic development was studied in plant leaves.

## 2. Results

### 2.1. A Geochemical Assessment of the Growth Conditions of L. maritimus

**Figure 1:**
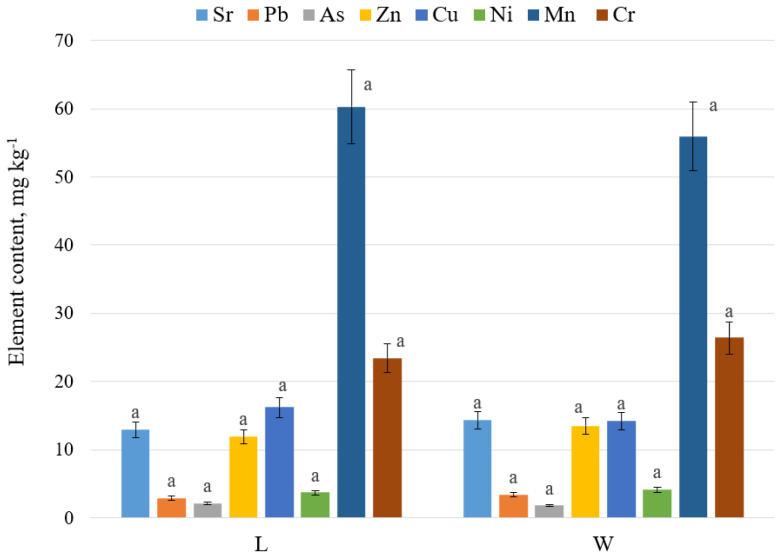
Element content in the accumulative soil horizon (0–10 cm) on the windward (W) and leeward sides (L) of the foredune, mg kg^−1^ dry weight (DW), July 2018. Data are expressed as mean values ± standard deviations, (n = 45). Letters indicate statistical difference; means with the same letter are not statistically different (Student’s *t*-test, *p* < 0.05).

Figure 1 shows the content of heavy metals in the sand samples of the foredune in the Curonian Spit national park. The concentration of heavy metals in sands was at minimum value, which was within sanitary standards (maximum allowable concentrations—MAC). The levels of heavy metals in the soil samples of the protective dune were as follows: lead—2.9–3.4 mg kg^−1^ (32 mg kg^−1^ MAC); zinc—11.9–13.5 mg kg^−1^ (55 mg kg^−1^ MAC); nickel—3.7–4.1 mg kg^−1^ (20 mg kg^−1^ MAC); copper—14.2–16.2 mg kg^−1^ (33 mg kg^−1^ MAC); strontium—12.9–14.3 mg kg^−1^ (clarke in the soil—380 mg kg^−1^); arsenic—1.8–2.1 mg kg^−1^ (2.0 mg kg^−1^ MAC); chromium—23.4–26.4 mg kg^−1^ (Cr^3+^—100 mg kg^−1^ MAC); manganese—55.9–60.3 mg kg^−1^ (300 mg kg^−1^ MAC). The metal content on the windward and leeward sides of the foredune in the soil did not differ significantly (*p* ≤ 0.05).

**Figure 2:**
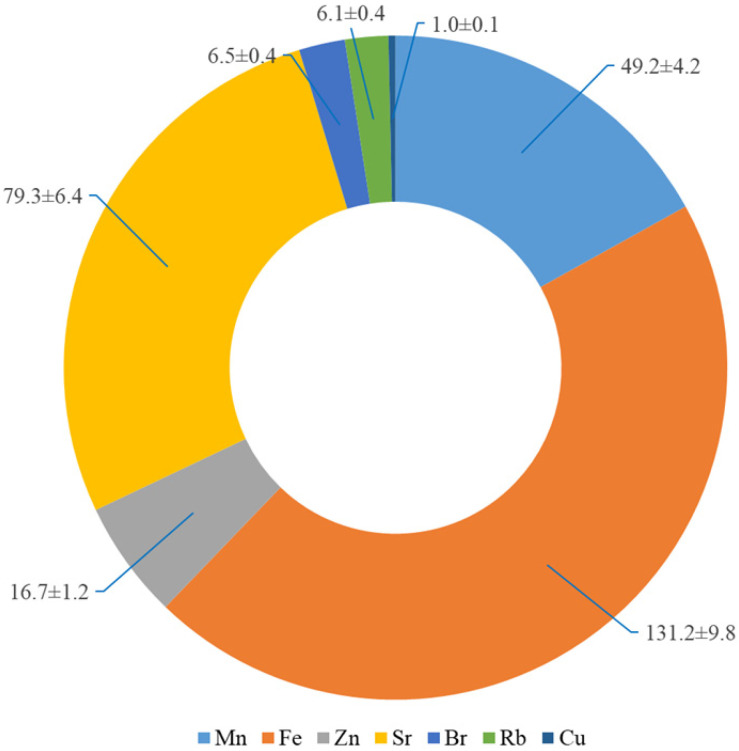
Content of elements in the leaves of *L. maritimus*, mg kg^−1^ DW (July 2018).

The accumulation of metals in the leaves of *L. maritimus* in the leeward side of the foredune did not differ significantly from the accumulation in plants growing on the opposite side of the dune (*p* ≤ 0.05). Figure 2 shows the average content of heavy metals in the leaves of each pea. Fe, Mn and Sr were prevalent in the leaves of the plant. The Pb level in the studied plants was about 0.9 ± 0.1 mg kg^−1^. The content of Ca in the plants was 0.01%.

### 2.2. Accumulation of Ascorbic Acid and Its Derivatives in Leaves of L. maritimus

**Figure 3:**
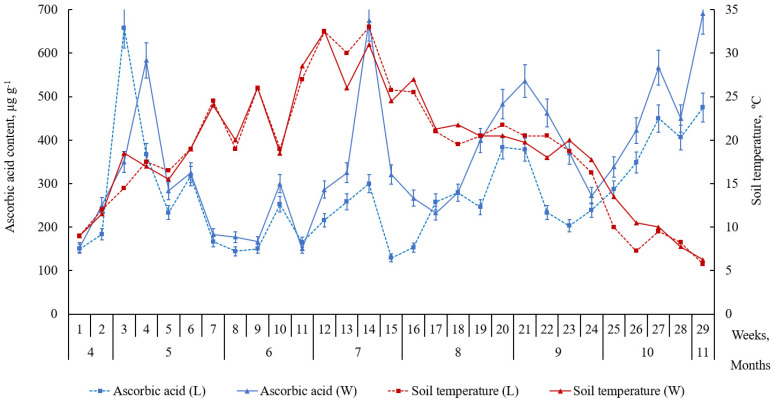
Content of ascorbic acid (AC) in the leaves of *Lathyrus maritimus* (μg g^−1^ DW) and temperature conditions of the leeward (L) and windward (W) sides of the foredune (April–November 2018). Data are expressed as mean values ± standard deviations, (n = 45). Differences were considered significant at *p* ≤ 0.05.

Our study of the content of ascorbic acid and its derivatives in the leaves of beach pea growing on the Curonian Spit shows that the level of AC (Figure 3) varied at different stages of ontogenesis. Accumulation peaks occurred at flowering stage, during increased soil temperatures in summer, and in late vegetative stage. The AC level at late vegetative stage was higher in the plants growing on the windward side of the foredune. The AC level drops after a peak at early vegetative stage. We observed intensive accumulation of reduced AC (675.8 ± 54.7 μg g^−1^) in week 14 of the study. Higher AC levels in the leaves of the plants growing on the windward side of the foredune were observed in weeks 18–21 and weeks 25–29 and reached its maximum of 691.7 ± 59.5 μg g^−1^. The accumulation of reduced AC in the leaves of beach pea growing on the leeward side of the foredune was very similar. The maximum AC level (658.3 ± 49.7 μg g^−1^) occurred in week three of the study. Later, the AC content decreased significantly and did not exceed the maximum value.

**Figure 4:**
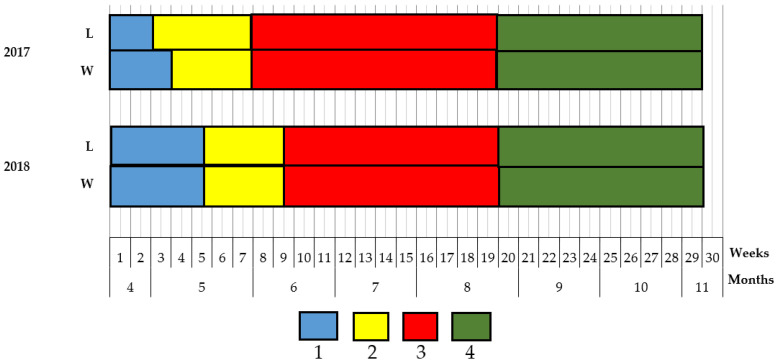
Phenological spectra of each pea on the leeward (L) and windward (W) sides of the foredune: 1—vegetation; 2—flowering; 3—fruiting; 4—dying.

Phenological observations demonstrated (Figure 4) that the plants growing on the leeward side of the foredune enters the above-discussed stages one-two weeks earlier than their counterparts on the windward side do. Thus, the peaks of AC accumulation in the leaves of beach pea growing on the leeward and the windward sides of the foredune occurred at different times, but differences appeared only at the flowering.

**Figure 5:**
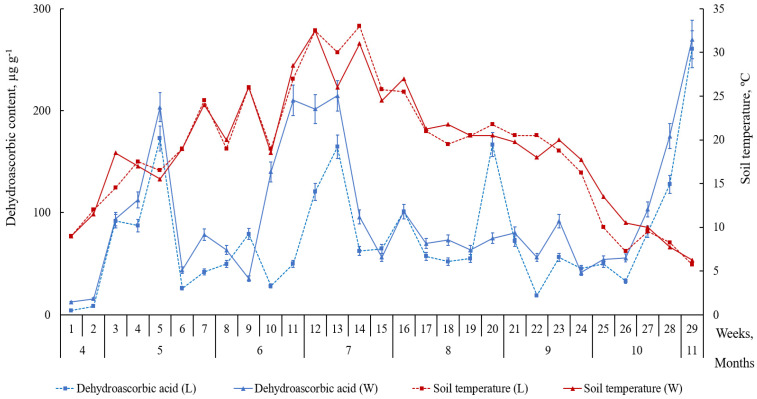
The content of dehydroascorbic acid (DAA) in the leaves of *Lathyrus maritimus* (μg g^−1^ DW) and the temperature conditions of the leeward (L) and windward (W) sides of the foredune (April–November 2018). Data expressed as mean values ± standard deviations, (*n* = 45). Differences considered significant at *p* ≤ 0.05.

**Figure 6:**
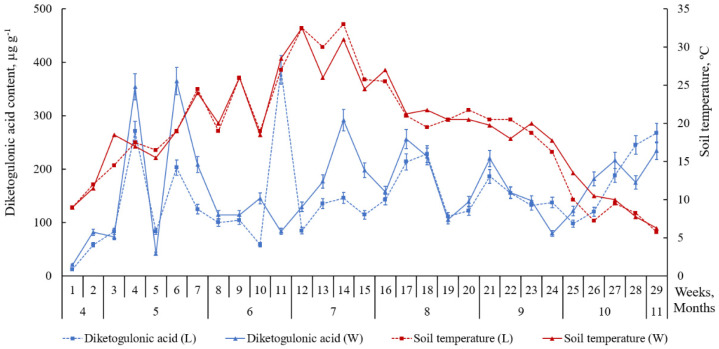
Content of diketogulonic acid (DKGA) in the leaves of *Lathyrus maritimus* (μg g^−1^ DW) and the temperature conditions of the leeward (L) and windward (W) sides of the foredune (April–November 2018). Data expressed as mean values ± standard deviations, (n = 45). Differences considered significant at *p* ≤ 0.05.

Similarly, to reduced AC, the content of oxidized AC forms (DAA and DKGA) was higher in the leaves of the plants growing on the windward side of the foredune (*p* ≤ 0.05). Moreover, it tended to increase during summer heatwaves (Figure 5 and Figure 6). Out of the three ascorbate system acids, the lowest level is associated with DAA, an oxidized form of AC (Figure 4). A small DAA content may suggest ongoing processes of DAA reduction to AC or its irreversible oxidization to DKGA. The level of reduced AC in the leaves was higher than that of oxidized forms (DAA and DKGA). The peaks of accumulation of the three acids in the leaves of beach pea alternate.

From week 26 of the study, a decrease in the soil temperature (below 11 °C) and the air temperature (below 13 °C) and stronger winds caused an increase in both the formation and consumption of ascorbic acid in the leaves of the plants. At the same time, we observed a growth in the content of its oxidized forms (DAA and DKGA) (Figure 5 and Figure 6).

**Figure 7:**
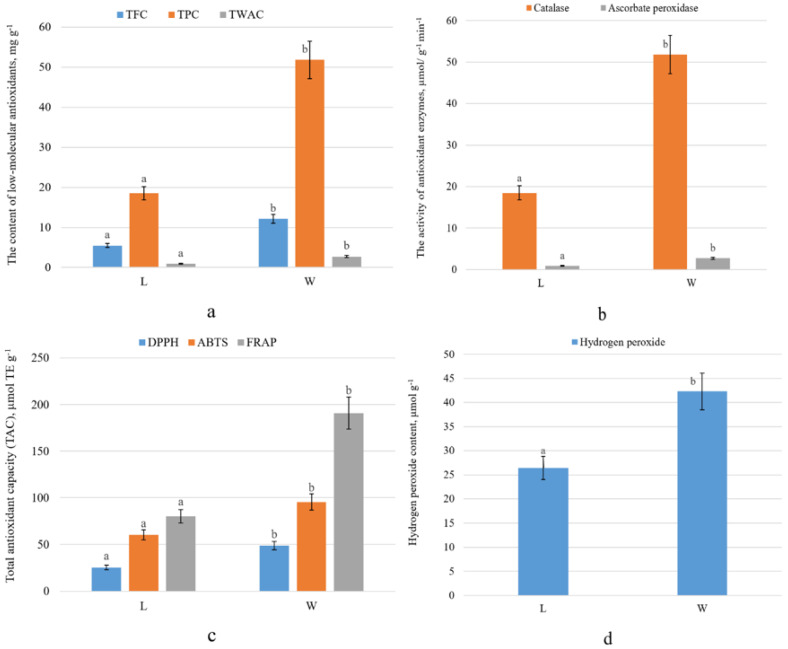
(**a**) Content of low-molecular antioxidants, (**b**) the activity antioxidant enzymes, (**c**) total antioxidant capacity and (**d**) the content of hydrogen peroxide in the leaves of beach pea on the windward (W) and leeward sides (L) of the foredune (July 2018). Data expressed as mean values ± standard deviations, (*n* = 45). Differences were considered significant at *p* ≤ 0.05. Letters indicate statistical difference; means with different letters considered statistically different (Student’s *t*-test, *p* < 0.05).

Figure 7a shows the total phenolics and flavonoids content in the leaves of beach pea. In the leaves of the windward side, phenols accumulate more actively: their content on this side was 2.8 times higher than that of the plants growing on the opposite side of the foredune (*p* ≤ 0.05). The content of flavonoids in the leaves of beach pea on the windward side of the foredune was 2.2 times higher than in plants on the opposite side of the foredune (*p* ≤ 0.05). Figure 7a shows the evaluation of antioxidant activity in beach pea. The total content of water-soluble antioxidants in the leaves of beach pea was higher in the plants of the windward side of the foredune (*p* ≤ 0.05). Another example of an active adaptation of beach pea to adverse environmental conditions is the increase in the pool of antioxidant enzymes. We have analyzed the activity of selected enzymes involved in the detoxification of hydrogen peroxide (catalase, ascorbate peroxidase) in the leaves of beach pea (Figure 7b). Higher enzyme activity was observed in the leaves of the plants on the windward side of the dune. There, the activity of catalase was 1.5 times, and the activity of ascorbate peroxidase 1.8 times higher than, that in the leaves on the leeward side of the foredune (*p* ≤ 0.05). The accumulation of hydrogen peroxide in plant tissues is shown in Figure 7d.

Antioxidant activity is a summary indicator and depends on the presence of a whole complex of compounds in the extracts. Figure 7c present the results of the antioxidant activity of *Lathyrus maritimus* extracts. In this study, three methods to evaluate the antioxidant activity of *Lathyrus maritimus* leaf extracts were employed (DPPH, ABTS, FRAP). To compare the data obtained by all three methods, we used Trolox as a standard in this work. The antioxidant activity of the beach pea extracts, regardless of the method of analysis (DPPH, ABTS, FRAP), was higher in plants on the windward side of foredune (*p* ≤ 0.05). Higher AOA values were obtained by the FRAP method. AOA of the leaves was 190.74 ± 8.7-μmol TE g^−1^ on the windward side and 80.12 ± 4.67-μmol TE g^−1^ on the leeward side of foredune.

## 3. Discussion

The decisive factor in the development of processes that violate the stability of natural plant complexes of the Curonian Spit is the wind. The concentration of heavy metals in sands is at minimum value, which is within sanitary standards (maximum allowable concentrations—MAC). In plants of *Lathyrus maritimus*, the concentration of metals also did not exceed phytotoxic values. Under these conditions, in addition to wind, a negative effect on plant growth and development can be a deficit of mobile forms of nitrogen, phosphorus and potassium compounds in foredune soils, low availability of humus and physical clay, as well as an increased amount of chlorides and sharp temperature difference in soil [15].

Stress-induced synthesis of low-molecular antioxidants (AC, glutathione, proline, polyamines, tocopherols, flavonoids, anthocyanins, carotenoids) has been extensively described in the literature [16,17,18,19]. AC is the most frequent low-molecular antioxidant in plants. Its concentration reaches 20 mM in the chloroplasts and can be high in the other compartments. This compound is an electron donor in many biochemical reactions. Many researchers have explored the role of AC as a low-molecular antioxidant [11,12,13,14]. Ascorbate is present in all plant cells, organelles and the apoplast. Ascorbate is synthesized in the mitochondria and reaches the other compartments along the proton electrochemical gradient and by diffusion. In the ascorbate–glutathione cycle, ascorbate peroxidase uses two molecules of ascorbate to reduce hydrogen peroxide to water with the formation of monodehydroascorbate. Monodehydroascorbate is an unstable radical that disproportionates to dehydroascorbate and ascorbate. The usual electron donor is NAD(P)H. Hydroascorbate reductase or ferredoxin catalyzes the reaction in the chloroplasts. Recent data suggest that ascorbate reacts with the hydroxyl radical, the anion radical and singlet oxygen. Ascorbate enters into the reactions of regeneration of tocopherol and oxidized carotenoids. This compound is most active in the process of detoxification of byproducts of redox reactions [11,12,13,14,16,19].

Our study of the content of ascorbic acid and its oxidized forms in the leaves of beach pea growing on the Curonian Spit shows that the level of AC (Figure 3) varied at different stages of ontogenesis. Accumulation peaks occurred at flowering stage, during increased soil temperatures in summer and in late vegetative stage. The AC level at late vegetative stage was higher in the plants growing on the windward side of the foredune.

AC is known to play a certain role in flowering and vegetative and reproductive differentiation [11,12,13,14,16,19]. Moreover, AC stimulates cell expansion and morphogenesis and facilitates blooming in some plants [11,12,13,14,16,19]. The level of AC increases dramatically at the beginning of flower differentiation [20]. The high content of AC in that period is attributed to the physiological need of an organism for metabolites necessary for the growth and development of plants [10,21,22].

The AC level drops after a peak at early vegetative stage. This decrease cannot be explained only by a reduction in the biosynthetic ability of leaves in that period. Obviously, it is associated with the rapid exhaustion of the metabolite during reproductive growth when a new type of metabolism develops. Moreover, reduced AC accumulation had a positive correlation with the soil temperatures (*r* = 0.63, *p* < 0.05). There is data to suggest that high temperatures induce the synthesis of ascorbate [10,22,23]. Probably, an increase in the AC content in the leaves is a response of the plants to high temperatures.

Similarly, to reduced AC, the content of oxidized AC forms (DAA and DKGA) was higher in the leaves of the plants growing on the windward side of the foredune. Moreover, it tended to increase during summer heatwaves. Out of the three ascorbate system acids, the lowest level is associated with DAA. A small DAA content may suggest ongoing processes of DAA reduction to AC or its irreversible oxidization to DKGA. The level of reduced AC in the leaves was higher than that of oxidized forms (DAA and DKGA). Therefore, reducing processes prevail over oxidizing processes or AC biosynthesis occurs at a higher rate than AC consumption. The peaks of accumulation of the three acids in the leaves of beach pea alternate. A reduction in the content of reduced AC is associated with an increase in the content of oxidized forms (DAA and DKGA). The above means that the complex redox processes in the AC ↔ DAA → DKGA system require significant time.

From week 26 of the study, a decrease in the soil temperature (below 11 °C) and the air temperature (below 13 °C) and stronger winds caused an increase in both the formation and consumption of AC in the leaves of the plants. At the same time, we observed a growth in the content of its oxidized forms (DAA and DKGA). There are data to suggest [5,24,25,26] that AC contributes to the resilience of plants to adverse conditions and low temperatures. Therefore, an increase in the level of AC in the leaves of beach pea in that period is a form of plant adaptation to cold. Another explanation of the active accumulation of ascorbate system acids is a growth in the content of glucose substrate in the plants [27,28,29]. Low temperatures are known to cause an increase in the concentrations of sugars (sucrose, glucose, galactose), which serve as a substrate for the biosynthesis of AC [27,28,29].

Adaptation to stress factors, particularly, low temperatures, consists in energy saving. The ability to maintain a high rate of photosynthesis while inhibiting other metabolic processes makes the low-temperature hardening of herbaceous plants possible [5,16]. It has been suggested [30,31,32] that, at lower temperatures, photosynthesis occurs at lower rates. Such conditions are associated with AC accumulation and lower consumption of the acid in beach pea at late vegetative stage. We observed a moderate negative correlation (*r* = −0.46/−0.68, *p* < 0.05) between the soil temperature and the accumulation of the ascorbate system acids in the leaves of the studied plants. During summer heatwaves, however, the correlation was positive (*r* = 0.54/0.63, *p* < 0.05).

We established that the AC, DAA and DKGA levels were higher in the plants growing on the windward side of the foredune. The foredune plants facing the sea have an increased content of both reduced AC and the oxidized AC forms (DAA and DKGA). Thus, AC consumption is more intensive in the plants of the windward side. AC can be used in photosynthesis, respiration and growth [11,12,13,14,19]. There are data to suggest that ascorbic acid contributes to the detoxification of the products of oxidative stress, which is caused by adverse environmental factors [11,12,13,14,19].

Our observations showed that the conditions of the windward side of the foredune were less favorable than those of the leeward side. The continuous mechanical effect of the wind from the sea, chloride salinization, low air temperatures, and decreased soil humidity can cause oxidative stress in plants. The activation of oxidizing processes increases the pool of AC and results in the accumulation of DKGA, a product of irreversible AC consumption. Thus, the plants adapt to the adverse conditions of the windward side of the foredune by active AC biosynthesis and consumption in the leaves of beach pea.

An important element of the antioxidant system is the fraction of phenolic compounds or polyphenols, which are among the most frequent secondary substances in plant cells. These compounds have aromatic (benzene) rings in their molecules (one in hydroxybenzoic and hydroxy–cinnamic acids and two in various flavonoids) and one or several hydroxyl groups bonded with the carbon atoms of an aromatic ring. The distinctive features of polyphenols are the universality of distribution, a wide range of variants (over 9000 compounds) and diversity of structures [9,33,34]. All of them are formed by the shikimic acid pathway and polyketide biosynthesis. Other distinctive features are easy oxidation accompanied by the formation of less-reactive transition products (semiquinone radicals or ortho-quinone), the ability of interact with proteins with the formation of hydrogen bonds and a tendency towards complexing with metal ions [9,33,34]. Polyphenols exhibit a high antioxidant activity, which is comparable to that of ascorbate and α-tocopherol [9,33,34]. The compounds are capable of inactivating free radicals and thus protecting cells against ROS.

Phenolic compounds generally accumulate in the vacuole and plastids. They contribute to photosynthesis, respiration, growth and development, fulfil mechanical, structural and signaling functions, and participate in the defense responses of plants to stress factors (including low temperatures) [35,36]. The antioxidant properties of phenols are a result of their ability to serve as electron donors in the peroxidase reaction and to take part in stabilizing unpaired electrons and the chelation of ions of transition metals [2,37].

The molecular composition of crude beach pea phenolic extracts is still unknown. In previous studies have demonstrated that *L. maritimus* served as an excellent source of condensed tannins (11.6 g/100 g) [38,39]. The extract of beach pea hulls exhibited a strong antioxidant activity in a β-carotene-linoleate model system. The extract contained different classes of phenolic compounds with varying antioxidative strengths. Further, showed the presence of (+) catechin and (−) epicatechin as the main low-molecular-weight phenolic compounds present. Thus, beach peas may present an alternate source of legumes for food use, while the pea hulls and their extracts could potentially be employed as nutraceutical ingredients [38,39].

Our studies have shown that phenolic compounds (TFC and TPC) accumulate more actively in the leaves of beach pea on the windward side of the foredune. A higher level of polyphenols and flavonoids in the leaves of beach pea on the windward side can compensate for the lack of endogenous low-molecular antioxidants (glutathione, ascorbate, proline, carotenoids, etc.), which is associated with the adverse conditions. This compensation is possible since phenols serve as electron donors in the peroxidase reaction [37].

Another example of an active adaptation of beach pea to adverse environmental conditions is the increase in the pool of antioxidant enzymes. We analyzed the activity of selected enzymes involved in the detoxification of hydrogen peroxide (catalase, ascorbate peroxidase) in the leaves of beach pea (Figure 7b). Higher enzyme activity was observed in the leaves of the plants on the windward side of the dune. There, the activity of catalase was 1.5 times and the activity of ascorbate peroxidase 1.8 times higher than, that in the leaves on the leeward side of the foredune. The activity of these enzymes in the leaves of the plants of the windward side is apparently caused by an increase in the content of peroxide in the tissues and the reinforcement of oxidizing processes (Figure 7d).

The formation of ROS in plant cell under stress has been considered as not only a damaging factor [1,2], but also the initial signal for the activation of the expression of genes taking part in adaptation to stress [12,25]. The process of complex transmission of stress signals involves superoxide (O^−^), hydrogen peroxide (H_2_O_2_) and hydroxyl radical (OH). At the onset of stress factors, the generation of superoxide anion radicals increases dramatically. As a response, the main enzyme that cleaves the radical, superoxide dismutase, becomes increasingly active. The superoxide anion radical, its derivative ROS peroxide compounds initiate and reinforce the chain processes of lipid peroxidation. Growing induction and increased accumulation of peroxide compounds inevitably lead to greater activity of catalase and ascorbate peroxidase, which are responsible for scavenging those compounds [12,40].

Ascorbate peroxidase and glutathione peroxidase are known to make a major contribution to antioxidant defense, which is associated with the reduction of hydrogen peroxide [12,40]. Ascorbate peroxidase is a heme-containing enzyme that uses ascorbic acid as an electron donor to reduce H_2_O_2_ [41]. The enzyme is generally localized in the chloroplasts (although it has been found in the mitochondria, the cytoplasm, the peroxisomes and the apoplast) and has a high affinity for hydrogen peroxide. Ascorbate peroxidase neutralizes even considerable concentrations of the former [42]. By taking part in the ascorbate–glutathione, ascorbate peroxidase decreases the content of H_2_O_2_, maintains redox balance in a cell and preserves the level of glutathione and ascorbic acid [43]. Highly specific to ascorbate, the enzyme rapidly becomes inactive in its absence [41].

Catalase takes an active part in scavenging hydrogen peroxide formed in plant cells. Remarkably, the enzyme scavenges hydrogen peroxide by energy saving. This process contributes to the preservation of reduced equivalents and energy in cells [42,44]. Although the reaction occurs at a high rate, catalase has a low affinity for H_2_O_2_ and activates at relatively high concentrations of the latter [42,44]. The literature has discussed the signaling role of H_2_O_2_ in the growth, morphogenetic and adaptive reactions in plants [42,44]. Excessive accumulation of ROS, however, is dangerous for cell plants because of its long lifetime and the ability to penetrate cell biomembranes [42,44]. AC, catalase and ascorbate peroxidase play an important role in the elimination of damage caused by oxidative stress in beach pea growing on the windward side of the foredune. The compounds contribute to the adaptive potential of the plant to an adverse environment.

All molecules having high Ox/Red potential can be inhibited (reduced) due to an antioxidant activity [45]. An antioxidant activity proves to be a summary indicator. It is dependent on the set of compounds found in plant extracts. At the present time, an antioxidant activity in plant extracts can be determined using a variety of methods. In addition, antioxidant action of these compounds appears to be different too. In this respect, the current study employs three assays of antioxidant activity evaluation in *Lathyrus maritimus* leaf extracts. An antioxidant activity of food products is measured using the DPPH and ABTS assays. These assays rest on the antioxidants ability to reduce the DPPH and ABTS radicals to nonradical forms. The FRAP assay is further used to assess an electron donating activity, which turns out to be a crucial mechanism of an antioxidant activity. This assay indicates the reducing power of the plant extracts. All the three mentioned above assays are employed to compare the data obtained. Trolox proves to be a standard in this work.

Another method for determining antioxidant activity is the amperometric method. The amperometric detection offers a number of advantages. These include a low detection limit, high selectivity (determined are only those compounds whose molecules can be oxidized, while other compounds are not determined, even if in high concentrations), a small volume of the electrochemical cell (0.1–5 μL) and easy servicing [46]. However, as shown in the study of Czyżowska et al. (2015) [47], when comparing antioxidant activity of wines from *R. canina* and *R. rugosa*, the measuring method should be taken into account. Different methods for determining antioxidant activity may show different results depending on the object of study [48].

A study of the antioxidant activity of *Lathyrus maritimus* leaves on the leeward and windward sides of the foredune showed that the content of water-soluble antioxidants (amperometric method) and antioxidant activity (DPPH, ABTS, FRAP) were higher in plants from the windward sides (*p* ≤ 0.05). There were also differences in the results obtained depending on the method of analysis—the highest antioxidant activity was obtained by the method (FRAP). There may be several reasons for the differences in the results of the antioxidant activity of *Lathyrus maritimus* extracts. First, DPPH and ABTS radicals are not absolute analogs of biologic radicals. Plant antioxidants can react very slowly with DPPH **^·^** and ABTS ^+^ or remain inert with them, and as a result, lower values of antioxidant activity [48]. In addition, the DPPH method is characterized by a nonlinear kinetic dependence of the reaction. This complicates the standardization of the determination method since it requires the selection of the reaction time depending on the specific sample [48].

## 4. Materials and Methods

### 4.1. Area and Species Characterization

**Figure 8:**
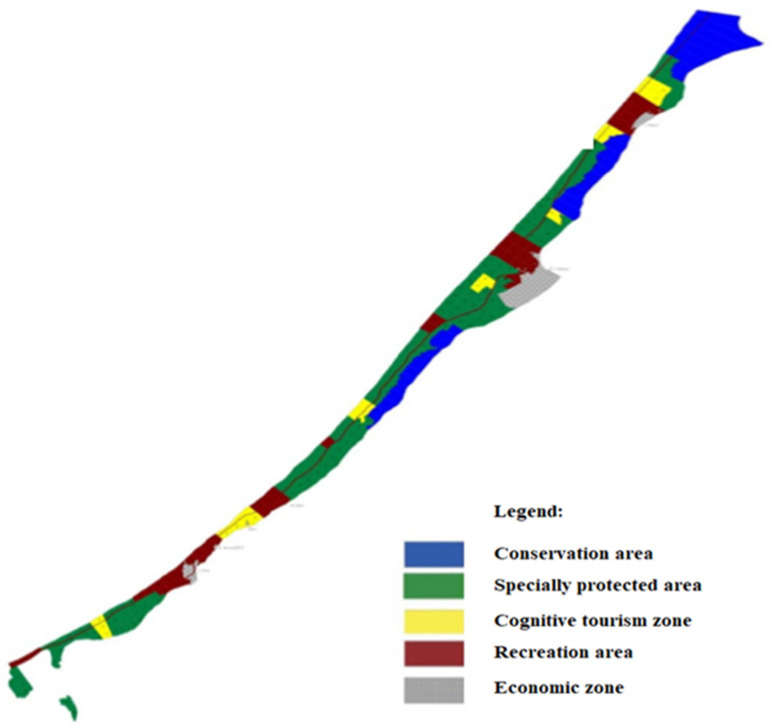
Functional zoning scheme of the Curonian Spit National Park [51].

The Curonian Spit is the largest aggradation body in the Baltic (Figure 8). The classification of the natural landscapes of the southeastern Baltic coast places the landform into a distinct type of the aeolian seacoast landscape. The shore terrain of the Curonian Spit is a sea beach with a protective dune ridge (foredune). The ridge varies in width (from 10–30 to 50–70 m) and height (from 3–7 to 10–15 m) and has a sharply asymmetric cross-section. The windward slope is steep, and the longer leeward slope is gradual [49,50,51,52].

The climate of the Curonian Spit is intermediate between marine and continental. The average annual temperature is +7.0 °C; the absolute minimum is −26 °C (January); the absolute maximum is +31 °C (June). The average annual precipitation is 660 mm. The period of peak precipitation is from October to February. The relative humidity is 80–90%. The key factor destabilizing the Curonian Spit ecosystem is the wind. Constant winds are a characteristic feature of the area. Throughout the year, it is dominated by SE, S, SW and W winds [49,50,51,52]. The foredunes and dune ridges have semi-fixed underdeveloped soils (they occupy 15% of the area). The humus content in these very thin (up to 20 cm) soils is insignificant, ranging from 0.2% to 0.3%. Many sections of the foredune and dune ridges, which are free of vegetation cover save sporadic annual herbs, are dominated by recent aeolian and windblown sands [49,50,51,52].

**Figure 9:**
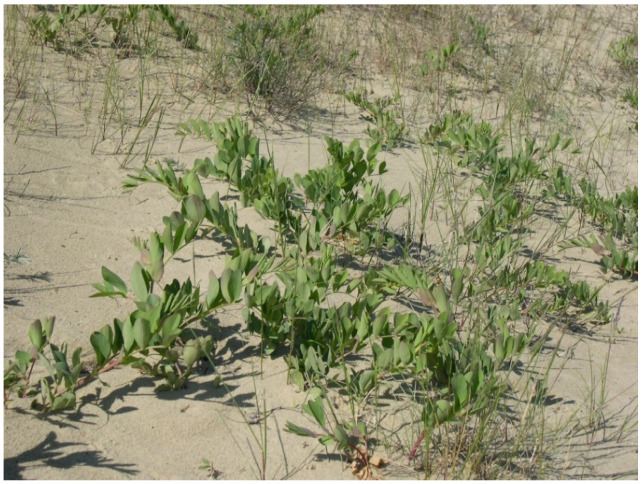
Beach pea (leeward side of the foredune). Phenological state: vegetative (photo by the authors).

The object of this study is beach pea (*Lathyrus maritimus* Bigel) growing on the windward (with reservations) and leeward sides of the Curonian Spit foredunes (Figure 9). It is a perennial greyish-green herb, growing to a height of 50–70 cm and having a creeping rhizome. The plant flowers in June–late August and bears fruit in September. The fruit is a large flat pod. The seeds are smooth, spherical and flattened [53].

### 4.2. Material Collection and Sample Preparation

The content of antioxidants and antioxidant enzymes was measured in the leaves of *L. maritimus*. The tests used the central part of the third from top open leaf. The tests were carried out weekly during vegetative stage from April to November in 2017–2018. Data are presented for the last growing season (2018). The antioxidant content was determined in fife mixed plant samples from three sites of the leeward (L) and windward (W) sides of the foredune in three analytical replicates (*n* = 45), the metal content in fife mixed plant and soil samples (*n* = 45). Samples of plants *L. maritimus* were taken in the areas of natural growth of this species. All selected plants were in good condition without any visible signs of mechanical and infectious damage. The plant material was collected from area, geographically located in the Curonian Spit National Park (specially protected natural area), belonging to the Zelenogradsky district of the Kaliningrad region of Russia. We identified the plant species using the “Illyustrirovannyy opredelitel’ rasteniy leningradskoy oblasti” and compared the collected material with samples of *L. maritimus* stored in the KLGU Herbarium. Ph.D. Maslennikov P. determined the herbaria specimens [53]. The soil samples were collected from the topsoil (epipedon) at a depth of 0–10 cm, using the envelope method [1,54]. The soils were analyzed at the collection sites, using standard techniques and measured air temperature and humidity, soil temperature and wind speed [1,54]. The content of the studied substances was measured by dry weight (DW). Preliminary sample preparation took place under laboratory conditions: the samples of plants were dried in a thermostat (Binder RE 115 Solid.Line, Germany) at a temperature of 60 °C, the samples of soils at a temperature of 105 °C to constant weight. The absolutely plant dry material was ground to the size of particles passing through a sieve with a hole diameter of 2 mm, soil dry material to the size of particles 71 microns.

### 4.3. Ascorbic, Dehydroascorbic and Diketogulonic Acid Content

The content of AC, DAA and DKGA were measured using a colorimetric technique [55]. One sample weight of the plant material (0.5 g) was ground with 25 mL of 1% metaphosphoric acid; another sample weight was ground with 25 mL 2·10^−3^-M unithiol solution prepared using a phosphate buffer. Centrifugation (20 min, 3000 RPM) was applied to separate out sediment. For protein pelleting, we added 4 mL of 1% of metaphosphoric acid to 16 mL of the centrifugate and centrifugated it for 15 min at 3000 RPM. To a 0.001-N solution of 2,6-dichlorophenolindophenol to a test tube were added containing 1.5 mL of the extract. Then, 0.5 mL of a 2% solution of 2,4-dinitrophenylhydrazine and 0.5 mL of the distillate product were poured into each test tube. The test tubes were placed inside a temperature-regulated chamber for 20 min at 100 °C. Then, 2.5 mL of 85% of sulfuric acid was added to the test tubes, waited an hour and performed photometric measurements at wavelength 520 nm. As control 3 mL of 1% metaphosphoric acid, 1 mL of 2,4-dinitrophenylhydrazine and 1 mL of the distillate product was used. The optical density of solutions was monitored using a Shimadzu UV3600 spectrophotometer (Shimadzu, Japan). The calibration curve was made by preparing AC solutions at different concentrations. In a test tube with an extract obtained with unitiol, the content of DKGA was determined. In a test tube with an acid extract, the content of DAA was determined. In a test tube with an acid extract obtained with 2,6-dichlorophenolindophenol, the ascorbic acid content was determined. The AC, DAA and DKGA content contents are expressed in μg of per gram dry weight, (μg g^−1^ DW).

### 4.4. Total Water-Soluble Antioxidants Content (TWAC)

The total water-soluble antioxidants content were estimated by an amperometric method using a TsvetYauza-01-AA (NPO Khimavtomatika, Inc., Moscow, Russia) according to Yashin [46]. The method consists in measuring the electric current generated when the analyte is oxidized on a working electrode surface at a certain potential. The technique applied made it possible to conduct a highly selective determination of soluble antioxidants in the samples. The sensitivity of the amperometric detector was very high (~10^−12^ A) because of low noise, whereas the detection limit of the device was at the nanogram to picogram level [46]. Plant extract preparation: 0.2 g of plant material was homogenized with 50 mL of eluent (solution of phosphoric acid with the molar concentration of 2.2 mM). The mixture was then filtered and used for analyses in the day of preparation. The calibration curve was made by preparing quercetin solutions at different concentrations. The total antioxidant contents are expressed in mg of as quercetin-equivalent (QE) per gram dry weight, (mg QE g^−1^ DW).

### 4.5. Total Phenolic Content (TPC)

To prepare the plant extract, a sample of 0.2 g plant material was homogenized in 10 mL of 96% (*v*/*v*) ethanol. Then, the extract was centrifuged at 4500× *g* for 30 min. The received supernatant was examined. The Folin–Ciocâlteu method [56] was employed to calculate total phenolic content. In the test tube, 100 µL of gallic acid standard or plant extract and 300 µL of 0.2-M Folin–Ciocâlteu reagent were mixed. The mixture was then incubated for 10 min in the dark at room temperature. Thereafter, 6 mL of 6.75% (*v*/*v*) sodium carbonate (Na_2_CO_3_) solution was poured into each test tube. The test tubes were incubated for 30 min in the dark at room temperature. Absorbance of the above-mentioned solution was recorded at 765 nm using the UV-3600 (Shimadzu, Japan). Total phenolic content was expressed in mg gallic acid equivalent per gram of dry weight (mg GAE g^−1^ DW).

### 4.6. Total Flavonoids Content (TFC)

To prepare the plant extract, a sample of 0.2 g plant material was homogenized in 10 mL of 96% (*v*/*v*) ethanol. Then, the extract was centrifuged at 4500× *g* for 30 min. The received supernatant was examined. Total flavonoid content (TFC) was determined by the colorimetric assay in accordance with the methodology proposed by Sevket et al. [57]. Into the 10-mL volumetric flask, 4 mL of dH_2_O, 100 µL of the plant extract, 0.3 mL of 5% sodium nitrite (NaNO_2_) solution and 300 µL of 10% aluminum chloride (AlCl_3_) solution were placed. In 6 min, 2 mL of 1-M NaOH solution was added into the flask. The total volume of the liquid in the flask was brought up to 10 mL with dH_2_O. Absorption of the reaction mixture was defined at 510 nm using the UV-3600 (Shimadzu, Japan). Total flavonoids content was calculated using a calibration curve and expressed as mg quercetin equivalent (QE) per gram of dry weight (QE g^−1^ DW).

### 4.7. Total Antioxidant Capacity (TAC)

To prepare the plant extract, a sample of 0.2 g plant material was homogenized in 10 mL of 96% (*v*/*v*) ethanol. Then, the extract was centrifuged at 4500× *g* for 30 min. The received supernatant was examined. Total antioxidant activity was measured using the DPPH (1,1-diphenyl-2-picrylhydrazyl) radical scavenging assay, the ABTS+ (2,2′azinobis(3)ethylbenzthiazoline-6-sulfonic acid) radical scavenging assay and the FRAP (ferric reducing antioxidant power) assay. Each extract was mixed with 2.85 mL of freshly prepared 0.1-mM DPPH solution in ethanol. The samples were incubated for 30 min in the dark at room temperature. Decrease in the solution absorption was measured spectrophotometrically at 515 nm using the UV-3600 (Shimadzu, Japan) [2]. The ABTS and FRAP assays were carried out as suggested by Taneva et al. [58]. The ABTS+ solution was obtained by mixing aliquots of an aqueous solution of 7.0 mM of ABTS and 2.45 mM of potassium persulfate. To perform an assay, 2.85 mL of the ABTS+ solution and 0.15 mL of obtained extracts were mixed. The mixtures were allowed to stand for 15 min in the dark at 37 °C, after which absorbance was measured at 734 nm using the UV-3600 (Shimadzu, Japan) against ethanol. The FRAP reagent was freshly prepared by mixing 10 parts of 0.3-M acetate buffer (pH 3.6), 1 part of 10-mM 2,4,6-tripyridyl–triazine (TPTZ) in 40 mM of HCl and 1 part of 20 mM of FeCl_3_ × 6H_2_O in dH_2_O. To start a reaction, 3.0 mL of FRAP reagent was mixed with 0.1 mL of the investigated extract. The reaction time was equal to 10 min. The reaction took place in the dark at 37 °C. Absorption of the solution was measured at 593 nm using the UV-3600 (Shimadzu, Japan) against a blank solution prepared with ethanol. All results of total antioxidant capacity measurement were expressed as μmol Trolox equivalents per gram of dry weight (μmol TE g^−1^ DW).

### 4.8. Ascorbate Peroxidase Activity (APA)

To determine ascorbate peroxidase (EC 1.11.1.11) activity, a sample of 1 g leaf material was homogenized in 10 mL of 50-mM phosphate buffer having pH 7.6 in the cold. Polyvinylpyrrolidone (0.3 g) was added to the suspension and the mixture was allowed to filter. Then, the resulting mixture was centrifuged at 12,000× *g* for 10 min. The reaction mixture contained 50 μL of 0.1-mM EDTA, 50 μL of 0.05-mM ascorbate, 50 μL of 0.1-mM hydrogen peroxide, 2.55 mL of 50-mM phosphate buffer having pH 7.6 and 300 μL of the plant extract which was obtained by homogenate centrifugation. Absorbance of the solution was measured at 290 nm using the UV-3600 (Shimadzu, Japan) against a control mixture devoid of the enzyme extract. Non-enzymatic ascorbate oxidation turned out not to be greater than 5%. Thus, its contribution was not taken into consideration. Decrease in absorbance during the first 30 s of the reaction was considered as a measure of the enzyme activity, which was expressed in µmol/(g^−1^ DW min^−1^) using the molecular extinction coefficient ε = 2.8 mM^−1^ cm^−1^ [59].

### 4.9. Catalase Activity

The activity of catalase (EC 1.11.1.6) was determined as follows. Leaf tissue (1 g) was ground with 10 mL of 50-mM phosphate buffer (pH 7.0). The homogenate was filtered and centrifuged at 8000× *g* for 10 min. The enzyme extract obtained (25 µL) was added to 2.9 mL of phosphate buffer (pH 7.0), and, immediately before the measurement, 90 µL of 3% hydrogen peroxide was added. A decrease in the optical density was measured at 240 nm for 1 min using the Shimadzu UV3600, Japan. The enzyme activity was calculated in µmol/(g^−1^ DW min^−1^) using a molecular extinction coefficient ε = 39.4 mM^−1^ cm^−1^ [59].

### 4.10. Hydrogen Peroxide Content

We determined the hydrogen peroxide content (H_2_O_2_) by a technique that uses hydrogen peroxide to oxidize Fe^+2^ ions to Fe^+3^, which form colored compounds with xylenol orange [60]. A sample weight of leaves (0.05 g) was homogenized in cooled acetone and centrifuged for ten minutes at 12,000× *g* at temperature 4 °C. Then, we added 1 mL of assay reagent (500-μM ammonium ferrous sulfate, 50 mMH_2_SO_4_, 200-μM xylenol orange and 200-mM sorbitol) to 1 mL of the supernatant and, waited 45 min and centrifuged it for five minutes at 10,000× *g*. Absorbance was measured at 560 nm using a Shimadzu UV3600 spectrophotometer. The concentration of hydrogen peroxide was derived from a calibration curve, using hydrogen peroxide solutions of concentrations ranging from 273 to 1500 ng/mL. The hydrogen peroxide content was expressed in μmol/g^−1^ dry weight.

### 4.11. Heavy Metals Analysis

The heavy metals concentration in the soil and plant samples was measured by the X-ray fluorescence method, using a Spektroskan Maks-G spectrometer (SPECTRON, Moscow, Russia). The operation principle of the spectrometer is as follows. The sample is treated with primary X-ray tube radiation. Then, the intensity of secondary fluorescent radiation from the sample is measured at wavelengths corresponding to the studied elements. Finally, the mass fraction of these elements is calculated using an earlier plotted calibration curve, which shows the dependence between the content of the studied element and the measured intensity. Spectrum lines are extracted using crystalline diffraction with subsequent scanning of the spectrum. Johansson X-ray optics system. The energy resolution on the FeKa line is 14–20 milliangstroms (45–65 eV). The power of the X-ray tube does not exceed four watts. The material of the X-ray tube anode is Mo (Ag, Cu), whereas the material of the analyzing crystal is lithium fluoride LiF, with crystallographic planes orientation, graphite. The detection limits of the studied elements were from one part-per-million. The upper limit of determination was limited by the maximum content of the element in the sample.

### 4.12. Statistical Analysis

The obtained data were statistically processed. Data were expressed as mean values ± standard deviations. The content of test substances was determined in five plant and soil samples from three sites of the leeward (L) and windward (W) sides of the foredune in three analytical replicates (n = 45). The statistical significance of differences between the variants was established using Student’s *t*-test. Differences were considered significant at *p* ≤ 0.05. A correlation analysis was conducted based on Pearson’s chi-squared test.

## 5. Conclusions

Stress-induced accumulation of low-molecular antioxidants (AC and its derivatives, flavonoids, polyphenols, flavonoids) and antioxidant enzymes in beach pea was explored in this work. We have examined the antioxidant activity of beach pea leaf extracts as a comprehensive indicator for assessing the total antioxidant content in plant tissues. It was established that the level of AC, DAA and DKGA was higher in the plants of the windward side of the foredune. The concentration of the acids in the leaves had several peaks associated with early vegetative stage, summer heatwaves and late vegetative stage. Out of the three acids, DAA had the lowest content. The small content of DAA may suggest ongoing processes of DAA reduction to AC or its irreversible oxidization to DKGA. The content of reduced AC was above that of oxidized AC forms (DAA and DKGA). Therefore, reducing processes prevail over oxidizing processes or AC biosynthesis occurs at a higher rate than AC consumption.

The growth conditions on the windward side of the foredune are less favorable than those of the leeward side. The continuous mechanical effect of the wind from the sea, chloride salinization, low air temperatures and decreased soil humidity can cause oxidative stress in plants. Growing induction and increase accumulation of peroxide compounds inevitably lead to greater activity of catalase and ascorbate peroxidase, which are responsible for scavenging those compounds. The plants adapt to the adverse conditions of the windward side of the foredune by accumulating low-molecular antioxidants (AC) crucial for the efficacy of ascorbate peroxidase, phenolic compounds (polyphenols and flavonoids), which contribute to scavenging peroxide and other water-soluble antioxidant metabolites.
